# Deep Learning Approaches to Detect Atrial Fibrillation Using Photoplethysmographic Signals: Algorithms Development Study

**DOI:** 10.2196/12770

**Published:** 2019-06-06

**Authors:** Soonil Kwon, Joonki Hong, Eue-Keun Choi, Euijae Lee, David Earl Hostallero, Wan Ju Kang, Byunghwan Lee, Eui-Rim Jeong, Bon-Kwon Koo, Seil Oh, Yung Yi

**Affiliations:** 1 Department of Internal Medicine Seoul National University Hospital Seoul Republic of Korea; 2 School of Electrical Engineering KAIST Daejeon Republic of Korea; 3 Sky Labs Inc Seongnam Republic of Korea; 4 Department of Information and Communication Engineering Hanbat National University Daejeon Republic of Korea

**Keywords:** atrial fibrillation, deep learning, photoplethysmography, pulse oximetry, diagnosis

## Abstract

**Background:**

Wearable devices have evolved as screening tools for atrial fibrillation (AF). A photoplethysmographic (PPG) AF detection algorithm was developed and applied to a convenient smartphone-based device with good accuracy. However, patients with paroxysmal AF frequently exhibit premature atrial complexes (PACs), which result in poor unmanned AF detection, mainly because of rule-based or handcrafted machine learning techniques that are limited in terms of diagnostic accuracy and reliability.

**Objective:**

This study aimed to develop deep learning (DL) classifiers using PPG data to detect AF from the sinus rhythm (SR) in the presence of PACs after successful cardioversion.

**Methods:**

We examined 75 patients with AF who underwent successful elective direct-current cardioversion (DCC). Electrocardiogram and pulse oximetry data over a 15-min period were obtained before and after DCC and labeled as AF or SR. A 1-dimensional convolutional neural network (1D-CNN) and recurrent neural network (RNN) were chosen as the 2 DL architectures. The PAC indicator estimated the burden of PACs on the PPG dataset. We defined a metric called the confidence level (CL) of AF or SR diagnosis and compared the CLs of true and false diagnoses. We also compared the diagnostic performance of 1D-CNN and RNN with previously developed AF detectors (support vector machine with root-mean-square of successive difference of RR intervals and Shannon entropy, autocorrelation, and ensemble by combining 2 previous methods) using 10 5-fold cross-validation processes.

**Results:**

Among the 14,298 training samples containing PPG data, 7157 samples were obtained during the post-DCC period. The PAC indicator estimated 29.79% (2132/7157) of post-DCC samples had PACs. The diagnostic accuracy of AF versus SR was 99.32% (70,925/71,410) versus 95.85% (68,602/71,570) in 1D-CNN and 98.27% (70,176/71,410) versus 96.04% (68,736/71,570) in RNN methods. The area under receiver operating characteristic curves of the 2 DL classifiers was 0.998 (95% CI 0.995-1.000) for 1D-CNN and 0.996 (95% CI 0.993-0.998) for RNN, which were significantly higher than other AF detectors (*P*<.001). If we assumed that the dataset could emulate a sufficient number of patients in training, both DL classifiers improved their diagnostic performances even further especially for the samples with a high burden of PACs. The average CLs for true versus false classification were 98.56% versus 78.75% for 1D-CNN and 98.37% versus 82.57% for RNN (*P*<.001 for all cases).

**Conclusions:**

New DL classifiers could detect AF using PPG monitoring signals with high diagnostic accuracy even with frequent PACs and could outperform previously developed AF detectors. Although diagnostic performance decreased as the burden of PACs increased, performance improved when samples from more patients were trained. Moreover, the reliability of the diagnosis could be indicated by the CL. Wearable devices sensing PPG signals with DL classifiers should be validated as tools to screen for AF.

## Introduction

### Background

Atrial fibrillation (AF) is the most common cardiac arrhythmia in clinical practice [1]. The prevalence and incidence of AF have risen over the years with the aging population [2]. The gold standard used to diagnose AF is the electrocardiogram (ECG) [3]. However, many patients with AF present paroxysmal symptoms or are asymptomatic; thus, the limited accessibility of the ECG during the symptom could lower the detection rate of AF. It is important to detect AF regardless of symptoms because asymptomatic patients with AF could present with stroke at their first manifestation [4]. AF is one of the major causes of stroke and its severity is worse in patients with AF than those without [5]. Therefore, it is important to detect AF in a potentially high-risk population who might benefit from stroke prevention with adequate anticoagulation control [6-9].

Recently, photoplethysmography (PPG) has been studied for long-term monitoring of AF, because of the ease of use and their utilization using wearable and mobile devices [10-12]. PPG monitoring incorporated into wearable technologies might permit improvements in the detection rate of AF in high-risk patients. Past studies using PPG for AF detection relied on predetermined feature extractions, for example, root-mean-square of successive difference of RR intervals (RMSSD) with Shannon entropy (ShE) and machine learning techniques such as the support vector machine (SVM), artificial neural network, and k-nearest neighbor [11,13]. The accuracy, sensitivity, and specificity for differentiating AF from a *clean* sinus rhythm (SR; ie, SR without any premature atrial complexes or PACs) of these methods were promising [14-18]. However, PACs were frequently exhibited in patients with paroxysmal AF or in those after successful cardioversion [19-21], which rendered AF detection using PPG from *unclean* SRs less practical. Low accuracy in differentiating AF from SR with PACs in the feature extraction step was a major limitation of previous approaches [22,23]. More sophisticated AF detection algorithms should be designed to render PPG monitoring more pragmatic.

### Objective

We aimed to develop deep learning (DL) classifiers using PPG as an input to distinguish AF from SR in the presence of PACs. We also suggested a method to compute a confidence level (CL) [24,25] for each decision in tested samples so that physicians could quantify the reliability of the results from the DL classifiers.

## Methods

### Study Population and Data Acquisition

This was a prospective, single-center study including patients with persistent AF admitted for elective DCC from September 2017 to April 2018. A total of 81 consecutive patients were enrolled. After verifying AF with 12-lead ECG, baseline PPG signals were collected over a 15-min interval by attaching a pulse oximeter to the patient’s nondominant arm’s index finger in the supine position. In addition, a single-lead ECG signal was acquired simultaneously to confirm the rhythm and was used as the gold standard. DCC (biphasic 100 to 200 J) was performed under light sedation after the baseline recording. Among 81 patients with DCC, 5 patients could not be converted to SR and 1 patient had improper data acquisition because of inappropriate bandwidth filters and sampling rate. In total, 75 patients with successful DCC underwent post-DCC PPG and ECG recording for over 15 min using the same methods. PACs were also monitored during the post-DCC recording period. During both periods of the measurements, the subject was required to rest on the bed with a supine position such that potential motion artifacts could be minimized. In total, 3 cardiologists interpreted the single-lead rhythm strips and verified the PACs and other atrial tachyarrhythmia. If there was a discrepancy between readings, then the senior electrophysiologists (EKC and EL) interpreted the rhythm and determined the final conclusion for the rhythms. We applied bandwidth filters (0.2 to 18 Hz) on both PPG and ECG data and then exported them in XML format for the DL training. The study protocol was approved by the Seoul National University Hospital Institutional Review Board and adhered to the Declaration of Helsinki.

### Dataset Manipulation and Deep Learning Framework

We constructed PPG samples for training and testing from the PPG recording data of 75 patients. Each patient’s 15-min pre-DCC and post-DCC data were divided into multiple 30-second fragments with 20-second overlaps. We used a data augmentation technique to increase the number of samples [24]. Each sample was labeled as *AF* if it was generated before the DCC and as *SR* if generated after the successful DCC. The detailed dataset manipulation method is presented in Multimedia Appendix 1.

**Figure 1:**
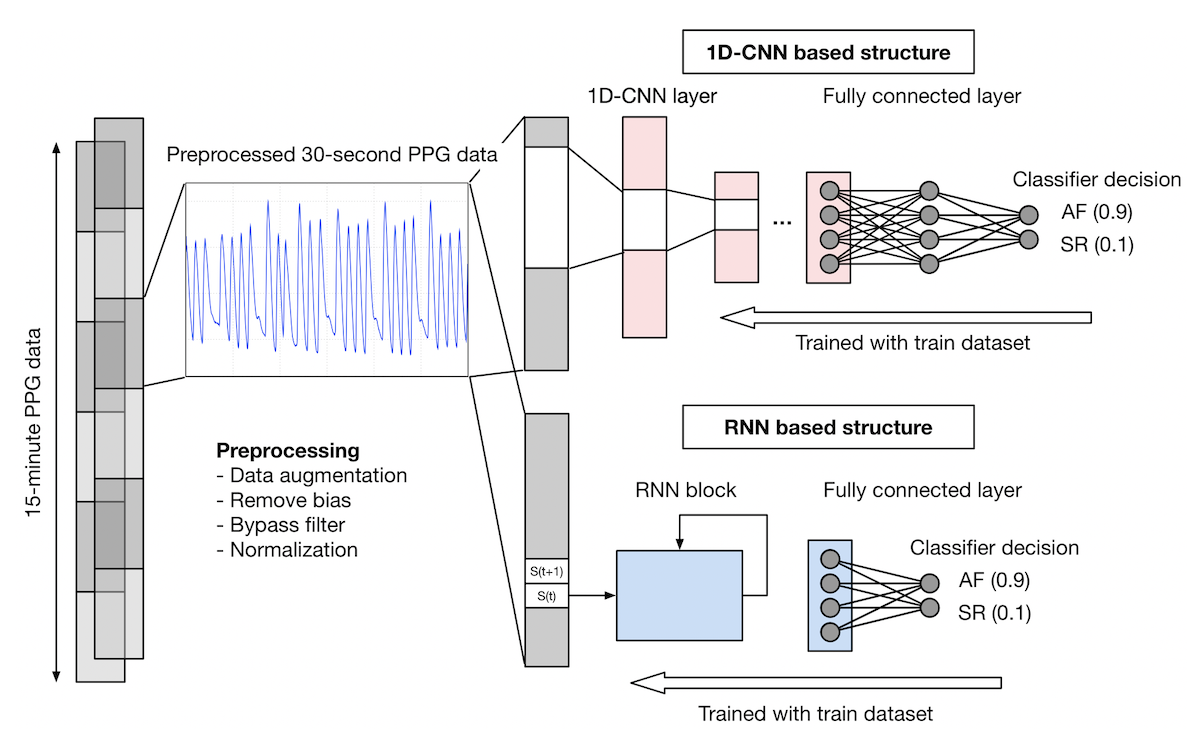
Flowchart illustrating the deep learning process. For each subject, 15-min PPG data during pre- and post- direct-current cardioversion periods were obtained. Each 15-min sample was preprocessed by removing bias, applying bypass filters, and normalization. Then, each sample was subdivided into 30-second samples with 20-second overlaps for data augmentation. The 30-second samples were trained and tested by 1-dimensional convolutional neural network (1D-CNN) and recurrent neural network (RNN) methods. Each sample was labeled as atrial fibrillation (AF) or sinus rhythm (SR). The number in parenthesis shows an example of the corresponding confidence level. In this example, the confidence level for diagnosing AF was 0.9 in 1D-CNN and RNN models, whereas 0.1 in SR for both models. 1D-CNN: 1-dimensional convolutional neural network; AF: atrial fibrillation; PPG: photoplethysmography; RNN: recurrent neural network; SR: sinus rhythm.

The entire DL framework of AF diagnosis is described in Figure 1. We used a 1-dimensional convolutional neural network (1D-CNN) and a recurrent neural network (RNN) as 2 DL architectures and compared their diagnostic performance. The DL process was divided into 2 phases: training and testing. The DL classifier consisted of several layers of artificial neurons, forming a kind of function approximator, simulating the neural connections of human brains. The neural network (NN) was trained to approximate a target function, which was the function of the 30-second-long PPG sample as input and the diagnostic decision as output. In the training phase, we trained the NN based on a supervised method that minimized differences between the AF and SR true labels and the NN outputs using a back-propagation algorithm. In the testing phase, we evaluated the trained NN classifiers with data that were not used in the training phase.

### Training and Testing Dataset Design

We used a 5-fold cross-validation approach to compare and evaluate 2 DL classifiers. The training phase included randomized features to initialize weight parameters between the NN nodes (weight and node corresponded to synapse and neuron, respectively) and the application of a rule to update weight parameters. Our 5-fold cross-validation process was as follows. In Scenario A, patients were randomly assigned to 5 groups, in which 4 were the training dataset, whereas the remainder was the testing dataset. Given that there were 5 groups of patients, 5 different combinations of the training and testing datasets were analyzed. In this scheme, no patients belonged to both the training and testing dataset at the same time, that is, DL classifiers always faced new patients during testing. We repeated the validation process over 10 times for each combination, and the final results were obtained by averaging the total of 50 validations. In Scenario B, we have performed 5-fold validation with random choices of samples, not in patients. Therefore, randomly chosen, 80% of the entire samples were assigned to training, whereas the remaining 20% were assigned to testing. Unlike Scenario A, Scenario B permits to allocate samples from the same patient into both training and testing datasets. We compared the performance with previous well-known AF detectors including SVM with RMSSD+ShE [15], SVM with autocorrelation [11], and an ensemble method by combining the 2 previous methods. Linear-kernel SVM was used in our study.

### Diagnostic Performance Using Different Algorithms

We compared the diagnostic performance of the different methods by generating receiver operating characteristic (ROC) curves. The area under the ROC curve (AUC) and the 95% CI for each method was calculated and compared using the DeLong test [26]. Statistical analysis was performed as a 2-sided test, and a *P* value less than .05 was considered statistically significant.

We also analyzed the accuracy (total number of true diagnosis of AF or SR divided by total number of test samples), sensitivity (the number of true diagnosis of AF divided by total number of AF-labeled test samples), specificity (the number of true diagnosis of SR divided by total number of SR-labeled test samples), positive predictive value (the proportion of AF-labeled test samples among the samples diagnosed as AF), and negative predictive value (the proportion of SR-labeled test samples among the samples diagnosed as SR). Each value was averaged over 50 validation processes for the 2 DL classifiers.

### Diagnostic Performance According to the Premature Atrial Complex Burden

We further analyzed the specificity of the DL classifier over the PAC burden in post-DCC rhythms. In Scenario A, the trained DL classifier faced new patients during the validation. Therefore, it is highly likely to encounter unknown samples during the testing phase. However, if the number of patients in the training set grows, even though the patients in the validation are new to the DL classifier, they will likely be similar to the patients seen in the training set. Scenario B emulates such a circumstance by making the sample distribution of the testing dataset similar to that of the training dataset. This could be achieved because the samples from the same patient could appear in both datasets. For each PAC burden and scenario, we compared a specificity by different algorithms. Then we evaluated whether DL classifiers outperformed previous algorithms over various PAC burdens and how they are improved by an assumption of the same sample distribution in both datasets (Scenario B).

### Confidence Levels

In this study, we defined the metric CL to measure the reliability of the diagnosis by a certain DL classifier. We refer readers to Multimedia Appendix 1 for a detailed description of the CL. The true CL represented the confidence of the classifier’s output when it has correctly diagnosed the patient (ie, AF as AF and SR as SR), whereas a false CL indicated the confidence when AF was diagnosed as SR or SR diagnosed as AF. The minimum CL was 50%, meaning that the diagnosis was randomly AF or SR, whereas the maximum CL was 100%, indicating that the diagnosis could be unquestionably AF or SR irrespective of the number of times the test was repeated.

## Results

### Baseline Characteristics of Study Population

A total of 75 patients (men 68/75, 91%; mean age 63 years, SD 7.8) were enrolled. Clinical characteristics of the study population are summarized in Table 1. A total of 18 patients (18/75, 24%) were long-standing persistent AF (AF history of >1 year). The median value of the CHA_2_ DS_2_-VASc score was 1.

### Characteristics of the Study Dataset

**Figure 2:**
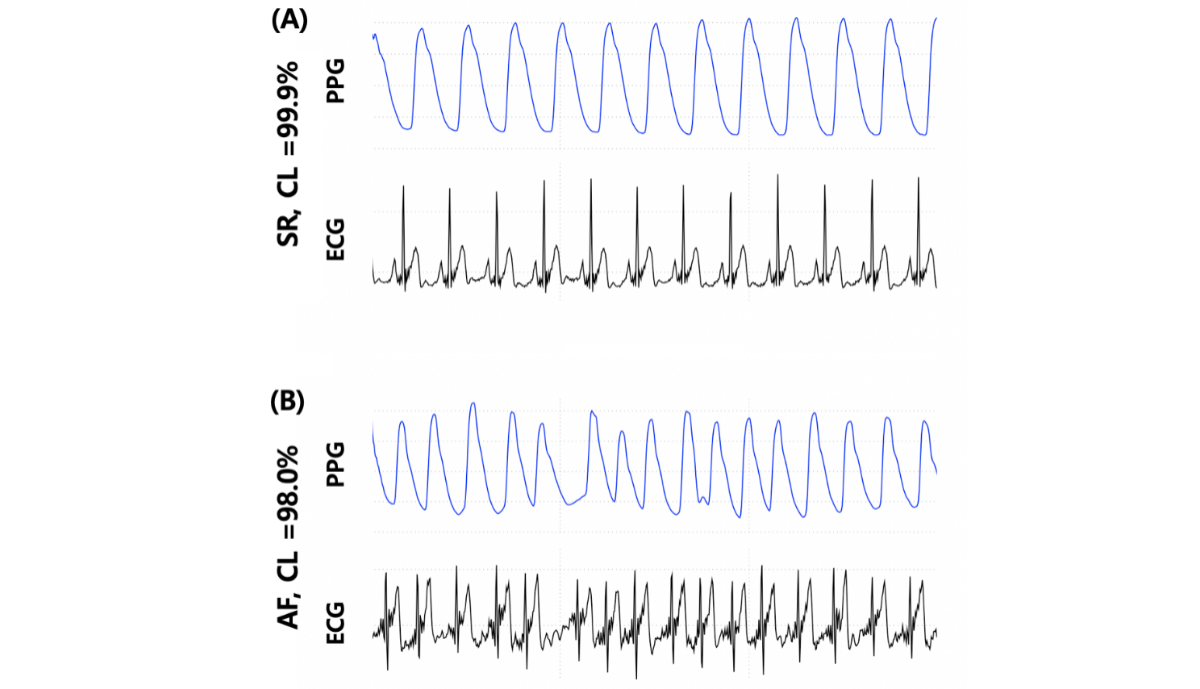
Typical examples of 15-second-long photoplethysmography and corresponding synchronized electrocardiogram samples for (A) stereotypic normal sinus rhythm and (B) atrial fibrillation with suggested confidence level using the 1-dimensional convolutional neural network algorithm. AF: atrial fibrillation; CL: confidence level; ECG: electrocardiogram; PPG: photoplethysmography; SR: sinus rhythm.

A total of 14,298 samples consisting of 30-second-long PPG were generated from the 75 patients. Each 30-second PPG sample was synchronized with a single-lead ECG to be diagnosed as AF or SR. Figure 2 shows examples of AF and SR determined by PPG recordings.

We developed a PAC indicator that could automatically detect the number of PACs in each post-DCC PPG sample to quantify the PAC burden of the study dataset. We applied a simple criterion: consider a beat as a PAC when the interval with the previous beat was less than 85% of the average interval. Multimedia Appendix 2 illustrates the PAC detection results of the indicator for a post-DCC PPG sample with 5 PAC episodes and the corresponding ECG signal. The proposed PAC indicator’s result was verified based on the cardiologist’s decision with a matched ECG signal. Both the PPG and the matched ECG samples were reviewed and the sensitivity and specificity of the PAC indicator after the review were 92.55% and 98.18%, respectively. The PAC burden of each post-DCC PPG sample was calculated by dividing the number of PAC with the number of beats in the sample (Multimedia Appendix 3). Inspection of the PAC indicator showed that 29.79% (2132/7157) of post-DCC samples contained PACs in their data.

The deep learning method may require considerable computational power and time during the training. However, because our NN structure was relatively lightweight because of a small number of layers (6 layers in 1D-CNN and 2 layers in RNN), the training phase in our study took about 10 min under the computational environment with a single GPU system using NVIDIA TITAN Xp graphics card.

### Comparison of the Performance of Deep Learning Classifiers

**Figure 3:**
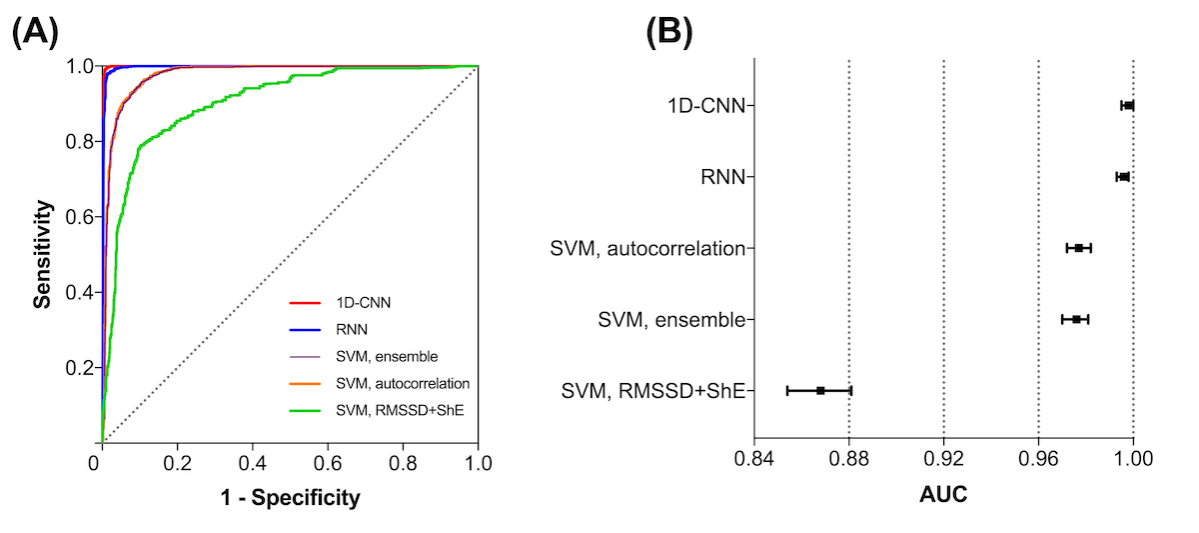
The receiver operating characteristic (ROC) curves of 2 deep learning classifiers (1-dimensional convolutional neural network, 1D-CNN and recurrent neural network, RNN) compared with other previous high-end atrial fibrillation (AF) detectors. (A) A Comparison of several ROC curves by different AF-detection algorithms. (B) The area under the curve and corresponding 95% CI by different algorithms. Both 1D-CNN and RNN methods showed significantly better diagnostic performance than previous detectors. 1D-CNN: 1-dimensional convolutional neural network; RMSSD: root-mean square of the successive differences of RR intervals; RNN: recurrent neural network; ShE: Shannon entropy; SVM: support vector machine.

The diagnostic performance of the DL classifiers with previous well-known AF detecting algorithms is summarized in Table 2. The results were obtained under Scenario A. The 1D-CNN and RNN showed high accuracy (97.58% [139,527/142,980] and 97.15% [138,912/142,980], respectively), sensitivity (99.32% [70,925/71,410] and 98.27% [70,176/71,410], respectively), and specificity (95.85% [68,602/71,570] and 96.04% [68,736/71,570], respectively). In addition, both methods showed high positive predictive values (95.98% [70,925/73,893] and 96.12% [70,176/73,010], respectively) and negative predictive values (99.30% [68,602/69,087] and 98.24% [68,736/69,970], respectively). We derived ROCs for all the algorithms and compared the different AUCs (Figure 3). The AUCs for 1D-CNN and RNN were 0.998 (95% CI 0.995-1.000) and 0.996 (95% CI 0.993-0.998), respectively, which were significantly higher than previous methods. The 1D-CNN and RNN showed larger AUCs compared with the SVM with RMSSD+ShE, SVM with an autocorrelation or ensemble method (all *P*<.001, compared using the DeLong test). There were no significant differences in the AUC for the 1D-CNN and RNN algorithms (*P*=.12).

In Scenario B, the performances of the DL classifiers improved in overall aspects but more in specificity and positive predictive value. Both 1D-CNN and RNN had improved sensitivity (99.71% [71,206/71,410] and 99.51% [71,059/71,410], respectively), specificity (99.35% [71,104/71,570] and 99.29% [71,062/71,570], respectively), positive predictive value (99.35% [71,206/71,672] and 99.29% [71,059/71,567], respectively), negative predictive value (99.71% [71,104/71,308] and 99.51% [71,062/71,413], respectively), and accuracy (99.53% [142,310/142,980] and 99.40% [142,121/142,980], respectively). Both classifiers had 1.000 of AUCs (95% CI 1.000-1.000).

**Table 1:** The clinical characteristics of the study population (N=75).

Variables		Values
**Demographics**		
	Age (years), mean (SD)	63 (7.8)
	Male, n (%)	68 (91)
	Body mass index (kg/m^2^), mean (SD)	25.2 (2.9)
	Body surface area (m^2^), mean (SD)	1.83 (0.16)
**Types of atrial fibrillation (AF), n (%)**		
	Persistent^a^	57 (76)
	Long-standing persistent^b^	18 (24)
CHA_2_ DS_2_-VASc score^c^		1 (1,2)
**Comorbidity, n (%)**		
	Congestive heart failure	5 (7)
	Hypertension	38 (51)
	Diabetes mellitus	10 (13)
	Stroke or transient ischemic attack	8 (11)
	Myocardial infarction	1 (1)
	Valvular heart disease	5 (7)
	Dyslipidemia	19 (25)
	Chronic renal failure	3 (4)
	Chronic obstructive pulmonary disease	0
	Hyperthyroidism	6 (8)
Previous AF ablation history, n (%)		3 (5)
**Antiarrhythmic agents, n (%)**		
	Propafenone	19 (25)
	Flecainide	9 (12)
	Pilsicainide	4 (5)
	Sotalol	1 (1)
	Amiodarone	40 (53)
	Beta blocker	18 (24)
	Calcium channel blocker^d^	12 (16)
	Digoxin	1 (1)
**Anticoagulant, n (%)**		
	Aspirin	2 (3)
	Warfarin	18 (24)
	Nonvitamin K oral anticoagulant	55 (73)
**Other medications**		
	Angiotensin converting enzyme inhibitor	0
	Angiotensin II receptor blocker	16 (21)
	Diuretics	7 (9)
	Statin	13 (17)

^a^AF history more than 1 month and less than 1 year.

^b^AF history more than 1 year.

^c^The value is expressed as both median and interquartile range.

^d^Nondihydropyridine class.

**Table 2:** The diagnostic performance of various algorithms for classifying photoplethysmography samples of atrial fibrillation and sinus rhythm after electrically cardioverted patients.

Algorithms	Accuracy	Mean sensitivity (%)	Mean specificity (%)	Mean positive predictive value (%)	Mean negative predictive value (%)	AUC^a^	95% CI	True mean confidence level (%)	False CL
1-Dimensional convolutional neural network	97.58	99.32	95.85	95.98	99.30	0.998	(0.995-1.000)	98.56	78.75
Recurrent neural network	97.15	98.27	96.04	96.12	98.24	0.996	(0.993-0.998)	98.37	82.57
Support vector machine, root-mean square of the successive differences of RR intervals + ShE^b^	86.82	89.13	84.50	85.16	88.63	0.868	(0.854-0.881)	—^c^	—
SVM, autocorrelation^d^	91.43	93.26	89.60	89.94	93.02	0.977	(0.972-0.982)	—	—
SVM, ensemble^e^	90.72	88.57	92.87	92.53	89.07	0.976	(0.970-0.981)	—	—

^a^AUC: mean area under the receiver operating characteristic curves. The standard errors by binomial exact test were all <0.01 except SVM with ensemble (0.01).

^b^SVM using RMSSD and ShE as a feature.

^c^Not applicable.

^d^SVM using autocorrelation method.

^e^SVM using RMSSD, ShE and autocorrelation.

### Performance of the Deep Learning Classifier and Burden of Premature Atrial Complexes

**Figure 4:**
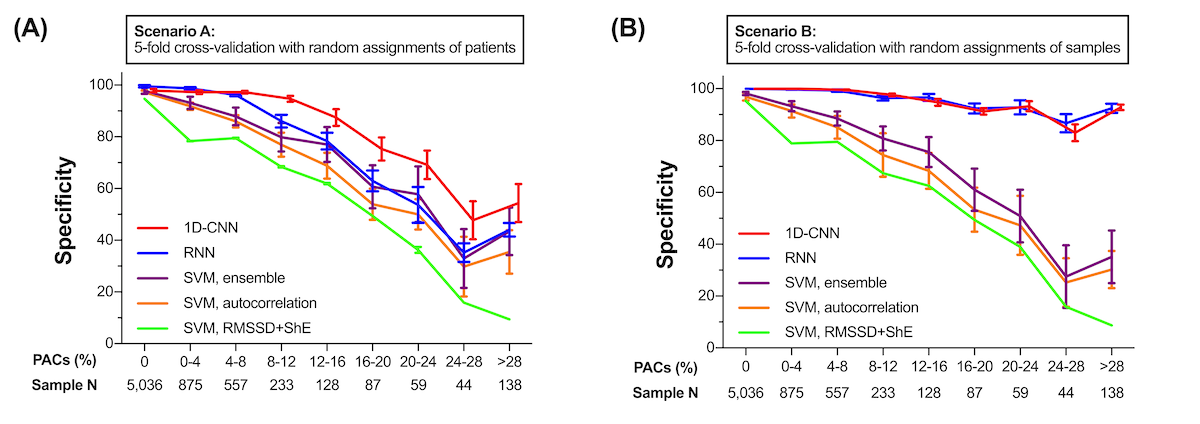
Comparison of performances of deep learning classifiers and previous state-of-the-art atrial fibrillation detectors by premature atrial complexes (PACs) burden. The performance of classifying photoplethysmography samples during post- direct-current cardioversion period as sinus rhythm by each algorithm was measured by specificity. (A) Scenario A was obtained by the 5-fold cross-validation with random assignment of patients. In this case, each algorithm faced new patient’s data during testing. (B) Scenario B was obtained by the 5-fold cross-validation with random assignment of samples. This approach assumed that the training distribution could emulate the test distribution. Regardless of the method, both 1-dimensional convolutional neural network and recurrent neural network maintained higher specificity over burden of PACs. Both DL classifiers showed higher specificity in Scenario B than Scenario A. 1D-CNN: 1-dimensional convolutional neural network; PAC: premature atrial complex; PPG: photoplethysmography; RNN: recurrent neural network root-mean square of successive difference of RR intervals.

Figure 4 shows the results with Scenario A and B. The burden of PACs was calculated for each 30-second PPG sample as the ratio of the number of PAC beats to the number of normal beats. The Sample N in Figure 4 shows the PAC burden distributions of the entire (both training and testing) dataset. In Scenario A, all the algorithms showed a decreasing tendency in specificity as the burden of PACs increased. Among the previous algorithms, the SVM with autocorrelation or ensemble maintained a higher specificity than with RMSSD+ShE. However, both 1D-CNN and RNN had a significantly higher specificity than the SVM with autocorrelation or ensemble (1D-CNN versus SVM with autocorrelation or ensemble, *P*<.001; RNN versus SVM with autocorrelation or ensemble, *P*<.001; all *P* values were calculated using a Student *t* test). Interestingly, the 1D-CNN maintained a significantly higher specificity than RNN in Scenario A (1D-CNN versus RNN, *P*=.02). In Scenario B, both DL classifiers improved significantly in specificity compared with Scenario A. The DL classifier could maintain 91.1% (1D-CNN) and 91.5% (RNN) specificity even for samples with a PAC burden ≥20%. Therefore, if the DL classifiers were trained with a sufficiently large dataset, they would maintain higher specificity even with a high PAC burden of and would outperform previous AF detectors.

### Confidence Level of the Deep Learning Classifier

**Figure 5:**
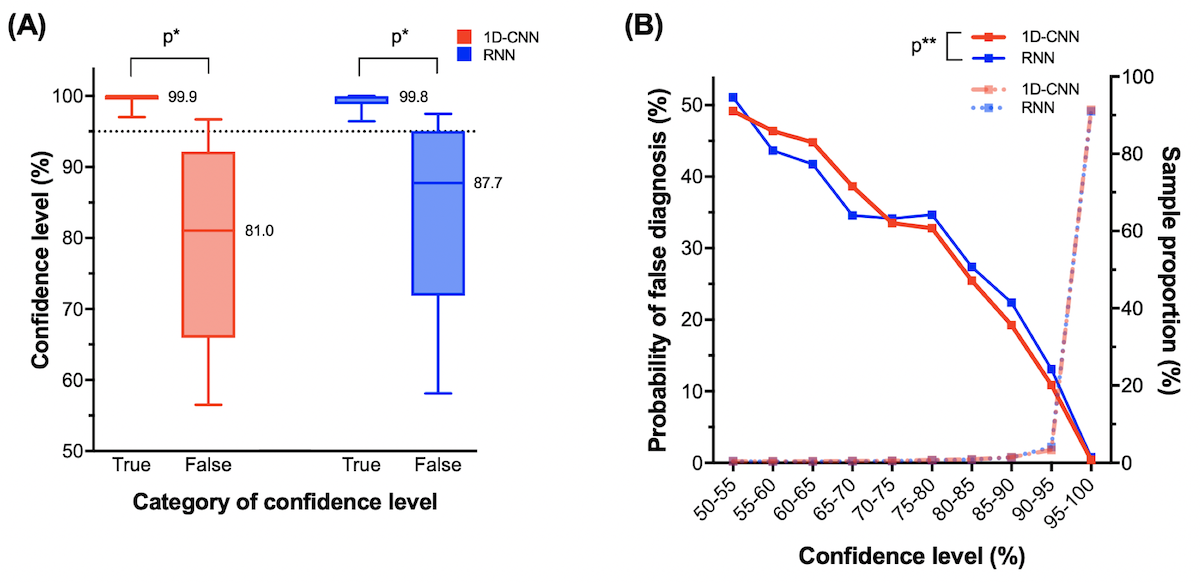
The characteristics of confidence level (CL) calculated by deep learning (DL) classifiers. Data were obtained by repeating the 5-fold cross-validation test over 10 times. (A) Comparison of true and false CLs of 1-dimensional convolutional neural network (1D-CNN) and recurrent neural network (RNN) methods by Box-and-Whiskers plot. True CLs indicate the cases where the diagnosis of a DL classifier was correct. Conversely, false CLs indicate cases where a DL classifier was incorrect. In the both 1D-CNN and RNN methods, the distributions of true or false CLs were significantly different (*P*<.001) for both 1D-CNN and RNN methods. If cut-off level of CL is to be 95% (dashed line), the diagnostic accuracy was 99.6% in 1D-CNN and 99.2% in RNN. Therefore, a diagnosis with a CL ≥95% can be regarded as certain. (B) The association between the probability of misdiagnosis and sample proportions and the respective CLs. Because 91% of the tested samples showed CL ≥95%, most diagnoses made by DL classifiers were valid. The probability of false diagnoses decreases from 50% to 0% as the CL increases from 50% to 100%. Comparing 1D-CNN and RNN, there was no significant difference in CLs (*P*=.98). *P<.001, calculated by Student *t* test. **P=.98, calculated by Student *t* test. 1D-CNN: 1-dimensional convolutional neural network; RNN: recurrent neural network.

Table 2 also shows true and false CLs of the 2 DL classifiers. The mean true and false CLs of the 1D-CNN classifier were 98.56% and 78.75%, respectively. With the RNN classifier, the true and false CLs were 98.37% and 82.57%, respectively. Therefore, significantly low CL values could indicate potential misdiagnoses. A further evaluation of the distribution of CLs is presented in Figure 5. For the 1D-CNN, the median values of true or false CL were 99.98% and 81.04%, respectively. Similarly, the median values for RNN were 99.81% and 87.74%, respectively. Comparison of the distribution of true or false CLs showed there were no significant differences in either the 1D-CNN or RNN methods (*P*<.001, calculated using a Student *t* test). If we set the cut-off level of CL to be 95%, the diagnostic accuracies were 99.58% (130,138/130,688) for 1D-CNN and 99.21% (129,053/130,082) for RNN. Therefore, a diagnosis with a CL ≥95% may be regarded as confident.

Even though DL classifiers were able to output a helpful metric, the classifier would become useless if most of the CL output were lower than 95%. However, 91.40% (130,688/142,980) of the tested samples had a CL >95% and the probability of a misdiagnosis for such sample was only 0.42% (550/130,688) for 1D-CNN. For RNN, likewise, 90.98% (130,082/142,980) of the tested samples had a CL >95% and the probability was 0.79% (1029/130,082). Therefore, most of the diagnosis made by the DL classifiers were confident. The differences in the probability of false diagnoses according to CL were not significantly different for both 1D-CNN and RNN (*P*=.98, calculated using a Student *t* test).

## Discussion

In this study, we developed a DL-based algorithm to diagnose AF using PPG data with better performance than previous algorithms. We found that (1) both 1D-CNN and RNN showed high diagnostic performance (AUC=0.998 and 0.996 for 1D-CNN and RNN, respectively); (2) both DL-based algorithms showed a better diagnostic performance than previous well-known AF detection algorithms, even under a high PAC burden, and had the potential to improve as more samples were allowed to be trained; and (3) most diagnoses by the DL classifiers were confident and the respective calculated CLs provided an easily interpreted reliability of the diagnosis.

### Previous Photoplethysmographic Signals–Based Atrial Fibrillation Detectors

Before the development of DL, several well-performing PPG-based AF detectors with algorithms, such as RMSSD+ShE with Poincaré plots [16], SVM with autocorrelation or with RMSSD+ShE [11,15] had been described that could detect the irregularity of intervals between each PPG pulse by utilizing explicit rules or features. A recent study showed that, among the selected non-DL algorithms, SVM performed better than any others including Poincaré plots [27]. However, most of these previous algorithms were based on explicit features regarding peak-to-peak intervals but not the information such as amplitudes or waveforms. This loss of information during feature extraction steps was a limitation of these algorithms. In this study, DL-based algorithms utilized the entire training data without any loss of information.

In contrast to the P-wave in ECG, the PPG has no markers for atrial contraction and this hinders the interpretation of cardiac rhythm from PPG alone. As a result, most previously developed algorithms for detecting AF from PPG relied heavily on the irregularity of peak-to-peak intervals. However, in the real-world setting, PACs are frequently observed in cardioverted AF patients and can simulate AF recurrence in these patients. Therefore, the diagnostic accuracy of PPG for detecting AF could be underpowered if the algorithm is predefined using a handcrafted approach. Therefore, more sophisticated methods to detect AF from PPG are needed.

### Novelty of Deep Learning Classifiers for Detection of Atrial Fibrillation

With regard to the outperformance of DL compared with traditional machine learning (ML), the following explanations should be considered. To solve a classification problem, as in our AF detection method, traditional approaches mainly rely on algorithms that are rule-based or handcrafted ML-based. However, discriminating AF from SR using PPG becomes much more challenging under the presence of high burden of PACs, as there are no P-waves in the PPG. Furthermore, the complexity increases as the frequency of the PACs grows, which often holds in practice. Although previous ML approaches rely on handcrafted features, which are extracted mainly based on human intuition, the DL analyzes all the characteristics of the trained data, which is not limited only to peak-to-peak intervals, but also contains waveform characteristics such as amplitude, frequency, and wave morphology, and then automatically and implicitly quantifies their significance. The DL is composed of multiple layers of NNs and their smart connections have proven to be a powerful and efficient tool to handle complicated problems via automatic feature extraction of data and an in-depth understanding of their correlations. The 1D-CNN and RNN are the NN architectures specialized in handling the sequential data. The 1D-CNN network is a set of learnable kernels to extract the specific features or patterns in the sequential data. The kernels are convolved across the time axis of the input sequence to compute more compressed output sequence. Multiple layers of 1D-CNN are stacked to get the fully compressed features from the input sequence. The RNN compresses the input sequence by performing the same feedforward operation for every input token with the output being dependent on previous operations. The information abstracted from the previous feedforwards is accumulated to the last operation so that the last output contains the fully compressed features. Unlike the 1D-CNN, we used only a single layer of RNN. This would explain how DL outperforms previously described algorithms. In addition to the higher detection accuracy achieved by DL, it is also able to provide an output of the Softmax probability for each decision, which is used to quantify the probability of a correct decision, that is, of the CL. These advantages of DL for higher detection accuracy and provision of the CL were significant for detecting AF using PPG as a dataset.

### Comparing Deep Learning Classifiers to Previous Algorithms

Recently, Poh et al reported that SVM showed the best performance among several non-DL algorithms and the deep convolutional NN was superior to SVM [27]. In our study, we compared 2 DL classifiers (1D-CNN and RNN) to previous well-known non-DL AF detection algorithms. The result showed that both DL classifiers had significantly better ROCs than previous methods including SVM and there were no significant differences between the ROCs of the 2 DL classifiers (Figure 3).

It should be noted that compared with previous studies, our results of both DL and non-DL algorithms showed fewer performances than expected. One may argue that our study should have had better results because we used a medical-grade pulse oximeter whereas some of the previous literature used sensors from smartphones, which would have a poorer signal-to-noise ratio [10,11,13,15,16]. However, such paradoxically lower results in our study may be explained by much higher PAC burdens in our samples (29.8%). Unlike other studies, we were able to graphically describe how diagnostic performances of various AF detectors degraded by PAC burdens (Figure 4). This implies any AF detector would give poorer results with more difficult samples, that is, the samples with more PAC burdens.

For DL classifiers, Poh et al reported better performance of CNN compared with this study (100.0% sensitivity and 99.6% specificity in the study of Poh et al but 99.3% and 95.9%, respectively, in our analysis) [27]. However, this can be explained by the different datasets used for training and testing. Our dataset was obtained from AF patients who underwent DCC. This scenario defines post-DCC rhythms with much more frequent PACs. Subsequently, the proportion of non-AF arrhythmia in the study sample was 6.4% in the study of Poh et al and 29.8% in our analysis. In other words, our training and testing dataset may be considered to be much more challenging in terms of AF or SR classification than those used in Poh’s study. Non-DL classifiers in our study also had lower performances than previous reports, but this may be again because of a much higher burden of PAC in our study. For example, with an SVM-based approach, Chan reported 92.9% sensitivity and 97.7% specificity for diagnosing AF from SR. However, the proportion of samples with PAC to those with non-AF was only 2.8% [11]. Therefore, the testing results of any algorithm heavily depend on the characteristics of the sample dataset. Nevertheless, as one can observe from Figure 4, the coherent superiority of the DL classifier’s performance over other algorithms for any PAC burden supports the advantages of using DL approaches to detect AF using PPG rather than previous non-DL algorithms.

Finally, there were other attempts to diagnose PAC from a normal SR or AF [16,28]. By incorporating RMSSD, ShE, and Poincaré plots, PACs were successfully diagnosed with 100% sensitivity and 97.8% specificity, yet information on how those results vary according to PAC burdens is lacking [28]. Similarly, such diagnoses would be performed by DL classifiers, provided that an investigator additionally labels the samples with PAC. Future research is needed to observe how DL classifiers are different from other algorithms when diagnosing not only AF but also PACs.

### Probability of Atrial Fibrillation Diagnosis

Besides the superior performance, the CL derived from DL classifiers was a useful metric to distinguish potentially mistaken decisions from correct decisions (Figure 5 and Multimedia Appendix 4). Multimedia Appendix 4 shows 2 exemplary cases where DL falsely diagnosed an AF but managed to generate low CLs (ie, 63.6% and 78.2%). However, according to Figure 5, such low CLs can be interpreted as *the diagnosis would possibly be incorrect* because the values are outliers to the lower 25th percentile of the distribution of true CLs. Thus, the possibility that the diagnosis in the examples would be correct would be approximately 35% to 45%. In such circumstances, the physician may attempt to confirm the cardiac rhythm using 12-lead ECG to validate the DL’s diagnosis. More simply, one may only accept the diagnosis by DL classifiers when the CL is ≥95%. We have described above how a diagnosis having such a high CL may be regarded as confident with an extremely low probability of misdiagnosis. An easy interpretation using the CL may be that *X%* of CL has an *X%* chance of true diagnosis by DL classifiers by observing the linearly decreasing probability of false diagnosis according to the CL (Figure 5).

In summary, our new DL classifiers showed not only better diagnostic performance of detecting AF compared with previous algorithms especially under the high burden of PACs, but were also helpful to physicians by suggesting the reliability of the diagnosis through CLs.

### Usefulness of Photoplethysmographic Signals for Atrial Fibrillation Detection

Most AF detectors rely on the ECG rather than on PPG because the interpretation of arrhythmia is more accurate and easier given the existence of the P-wave. However, in the clinical setting, monitoring the PPG rather than the ECG is easier and simpler. Furthermore, the emergence of wearable devices and mobile technologies enable clinicians to monitor the PPGs of a patient over long-term periods. Thus, the combination of AF detectors using PPG and wearable technology has the synergistic potential to screen AF recurrence and potentially to help prevent stroke in patients with AF. Though much effort has been made to utilize PPG to detect AF from SR, the presence of intermittent disruptions of regularity by frequent PACs hinder the application of PPG-monitoring for AF patients in real-life clinical situations. Future studies are needed to demonstrate that wearable devices sensing PPG to detect AF have clinical benefits in the prevention of stroke.

### Limitations

First, we did not diagnose other arrhythmias such as ventricular premature complex, atrial tachycardia, and sinus arrhythmia, but focused specifically only on PAC. As a result, DL classifiers designed in our study may not be applied to patients with other arrhythmias. However, this can be justified as most electrically cardioverted AF patients have more frequent PACs than other arrhythmias. Also, PACs have importance as a high burden is associated with AF recurrence [29]. Second, the training and testing datasets of this study consisted of PPG data from 75 patients. The total number of samples generated by data augmentation was 14,298, which was sufficient to train DL. Despite this number, 75 patients may be insufficient to capture AFs presenting different characteristics and PACs’ burden. Applying these trained DL classifiers to data from new patients may result in poorer performance. However, our analysis comparing scenario A and B (Figure 4) suggested that a sufficient number of samples from additional patients may result in improved performance. Finally, motion artifacts can arise during the measurement and cause performance degradation in a wearable device. To reduce the influence of motion artifact on the recording, we performed the signal in the supine position with minimal movement in our study. DL classifiers showed satisfactory performances without correction or removal of samples because of motion artifacts. However, we could not guarantee the performance of the developed deep learning algorithm in ambulatory patients would be as satisfactory as our results. Future works are needed to develop an algorithm that covers deleterious effects from motion artifacts.

### Conclusions

In this study, we developed DL classifiers to detect AF from SR under the presence of PACs using 30-second PPG samples during pre- and post-DCC periods from patients with AF. The diagnostic performances of 1D-CNN and RNN models were significantly superior to other previously well-known AF detectors. The diagnostic performance of this model was still better than previous algorithms even under frequent PACs, and we observed the potential for further improvement in performance when sufficiently larger samples could be trained. As a metric representing the reliability of diagnosis, the CLs can be calculated using DL classifiers and a diagnosis with a CL ≥95% may be considered as confident. Besides, most diagnoses determined by DL classifiers were found to be confident. Taken together, these advantages indicate that implementing DL classifiers to wearable devices sensing PPG signals can be useful for AF screening among patients with AF.

## Appendix

Multimedia Appendix 1 Detailed description of dataset manipulation, deep learning framework, and confidence level.

Multimedia Appendix 2 Exemplary comparison between single-lead electrocardiogram (ECG; upper panel) and simultaneous photoplethysmography (PPG; lower panel). Premature atrial complexes (PACs) in PPG found by PAC indicator (red dots) were well corresponded to true PACs observed in ECG. The green dots were normal peaks of PPG pulse calculated by the indicator.

Multimedia Appendix 3 The distribution of the post- direct-current cardioversion (DCC) photoplethysmography (PPG) samples used in the study subdivided by premature atrial complex (PAC) burden inspected by the PAC indicator devised in the study. Overall, 2132 out of total 7157 post-DCC PPG samples (29.79%) presented a PACs burden.

Multimedia Appendix 4 Exceptional examples of both photoplethysmography (PPG) and corresponding to synchronized electrocardiogram samples with frequent premature atrial complexes (PACs) mimicking atrial fibrillation (AF) and 1-dimensional convolutional neural network (1D-CNN) misdiagnosed as AF but able to generate low confidence level (CL) values. All samples were obtained during the post- direct-current cardioversion period. Asterisks denote PACs. (A) The case in which frequent PACs occurred and the CL was determined as 63.6%. (B) The presence of frequent PACs resulting in a couplet and with a CL of 78.2%.

## Abbreviations

1D-CNN: 1-dimensional convolutional neural network
AF: atrial fibrillation
AUC: area under the curve
CL: confidence level
DCC: direct-current cardioversion
DL: deep learning
ECG: electrocardiogram
ML: machine learning
NN: neural network
PAC: premature atrial complex
PPG: photoplethysmography
RMSSD: root-mean square of successive difference of RR intervals
RNN: recurrent neural network
ROC: receiver operating characteristic
ShE: Shannon entropy
SR: sinus rhythm
SVM: support vector machine
