# Supplementary material for: Deep Learning Approaches to Detect Atrial Fibrillation Using Photoplethysmographic Signals: Algorithms Development Study
Source: JMIR Mhealth Uhealth. 2019 Jun 6;7(6):e12770. doi: 10.2196/12770 (PMC6592499; doi:10.2196/12770)
Supplement: Multimedia Appendix 1 [file mhealth_v7i6e12770_app1.pdf]

Dataset Manipulation Method

During the process of sample generation, we allowed 20-second overlaps between samples, extracting a 30-second fragment with 10-second strides. Using this data augmentation method, approximately 180 samples were produced per patient, resulting in 14,298 samples in total as a dataset.

Deep Learning Structures

In this paper, we built two deep learning (DL) classifiers: 1- dimensional convolutional neural network (1D-CNN) and a recurrent neural network (RNN), two representative structures for learning sequential data. The 1D-CNN is a neural network that outputs the abstraction of the input without losing the sequential information. The suggested DL classifier utilizing the 1D-CNN consisted of six layers of artificial neurons. The bottom four layers were 1D-CNN layers and the top two layers were fully connected layers, which represent the basic type of artificial neural layers. RNN is a DL structure that greatly reduces the number of parameters by introducing the concept of memory while having a similar functionality as 1D-CNN. The suggested DL classifier utilizing RNN consisted of only two layers of artificial neurons, the Gated Recurrent Unit (GRU) at the bottom and a fully connected layer at the top. We used Google's Tensorflow framework to implement and evaluate the performance of the proposed classifier structures. The number of parameters in the 1D-CNN-based DL classifier was 6,431,365, and the number of parameters in the RNN based DL classifier was 85,507.

Confidence Level

The suggested neural network (NN) outputs the Softmax probability for each label, and we regarded the label with the maximum Softmax probability as the decision of the NN. The

maximum Softmax probability naturally indicates how much the NN believes the decision to be the correct answer. However, the confidence level, maximum Softmax probability, of modern models shows a higher average value compared to the average accuracy, meaning that the NN is overconfident at its decisions. In this study, we applied temperature scaling on the output Softmax probability to calibrate it as a more realistic measure of the confidence level. Temperature scaling was applied after the training step, as a post-processing procedure, which did not include accuracy.
