# Supplementary figures and images for: Deep Learning Approaches to Detect Atrial Fibrillation Using Photoplethysmographic Signals: Algorithms Development Study
Source: JMIR Mhealth Uhealth. 2019 Jun 6;7(6):e12770. doi: 10.2196/12770 (PMC6592499; doi:10.2196/12770)

## Slide 1
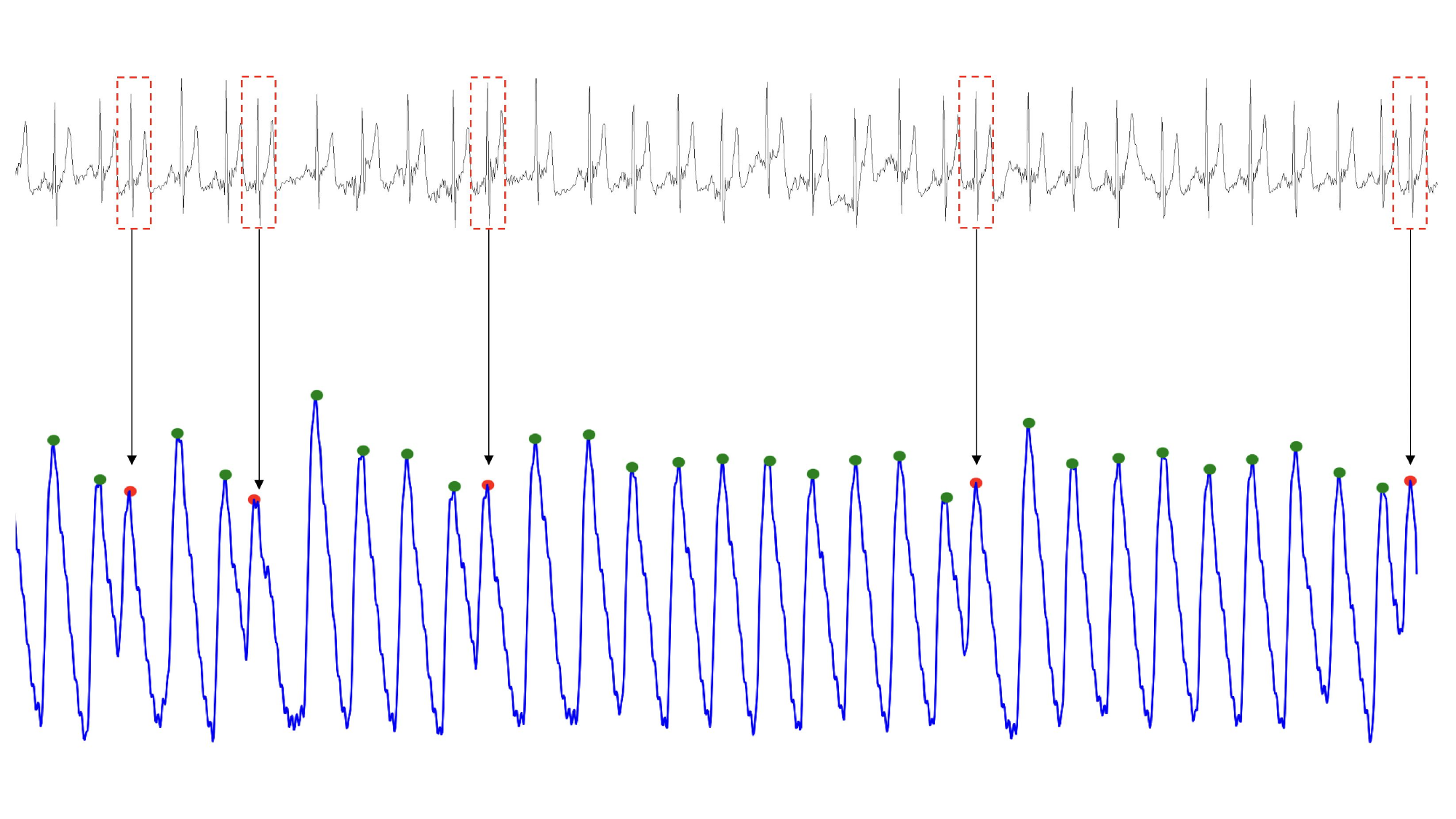

Supplement: Multimedia Appendix 2 [file mhealth_v7i6e12770_app2.pptx]

## Slide 1
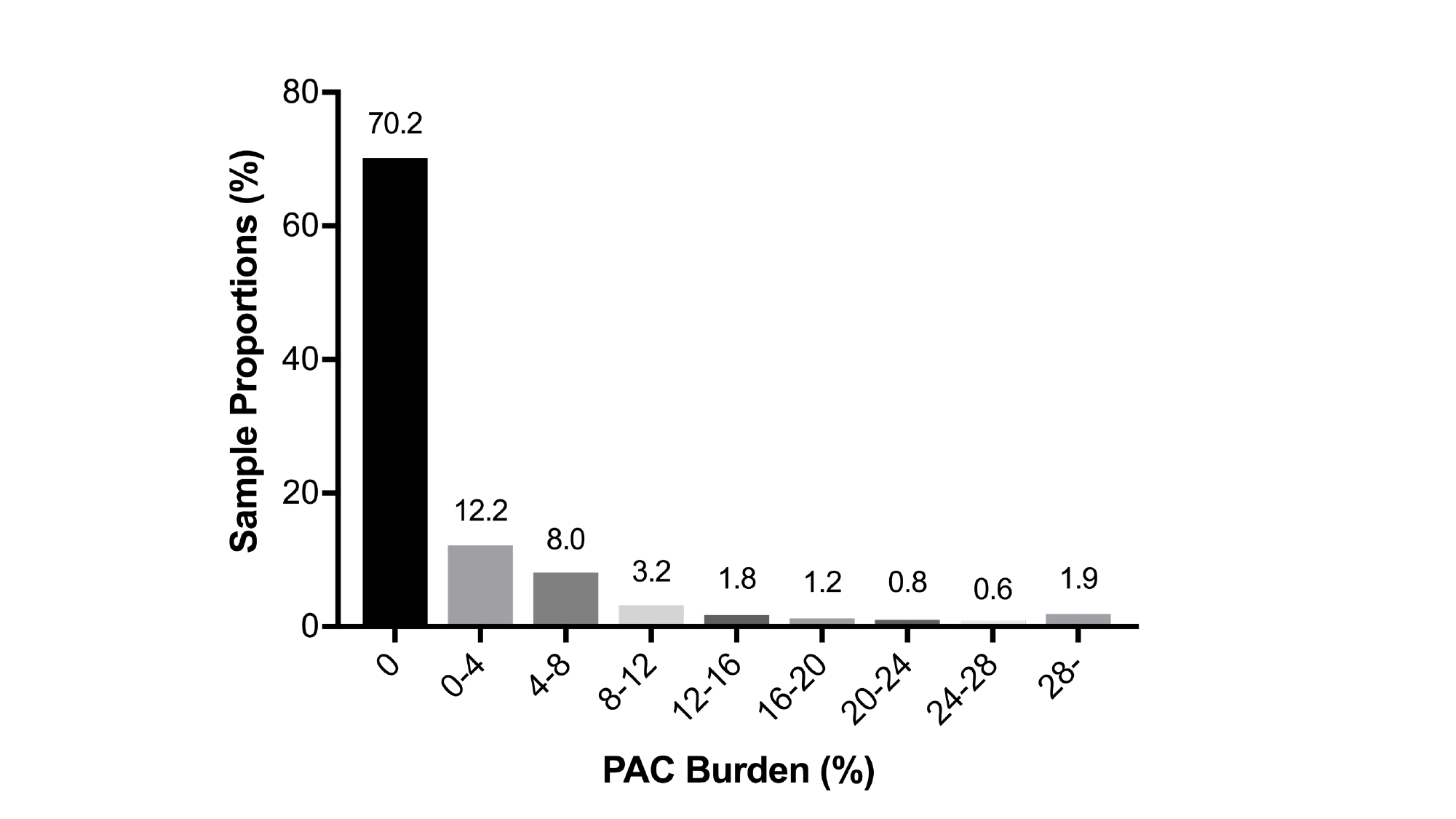

Supplement: Multimedia Appendix 3 [file mhealth_v7i6e12770_app3.pptx]

## Slide 1
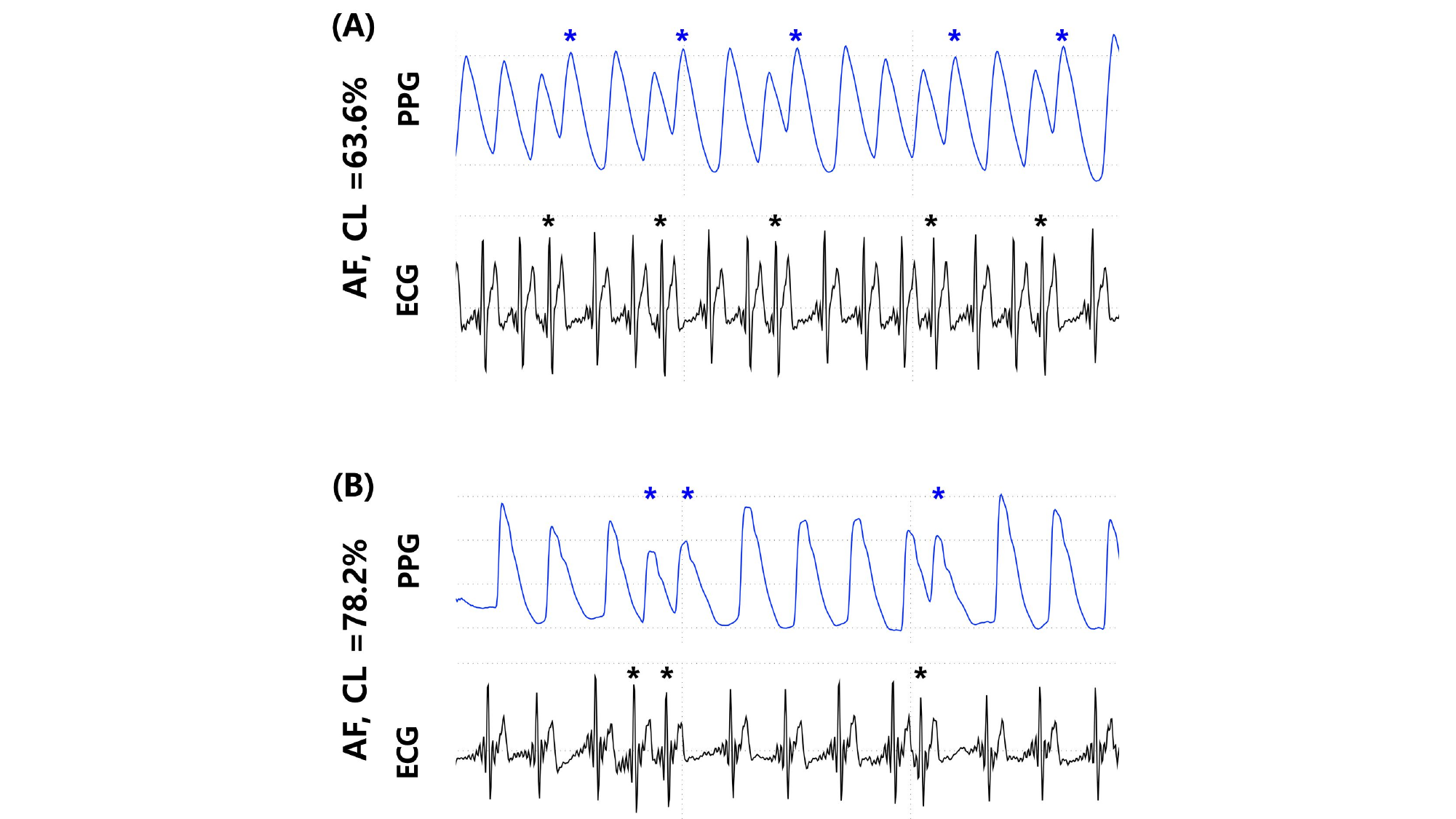

Supplement: Multimedia Appendix 4 [file mhealth_v7i6e12770_app4.pptx]
